# Liquid biopsy for diagnostic and prognostic evaluation of melanoma

**DOI:** 10.3389/fcell.2024.1420360

**Published:** 2024-08-02

**Authors:** Nicholas Slusher, Nicholas Jones, Taichiro Nonaka

**Affiliations:** ^1^ School of Medicine, Louisiana State University Health Shreveport, Shreveport, LA, United States; ^2^ Department of Cellular Biology and Anatomy, Louisiana State University Health Sciences Center, Shreveport, LA, United States; ^3^ Feist-Weiller Cancer Center, Louisiana State University Health Shreveport, Shreveport, LA, United States

**Keywords:** melanoma, liquid biopsy, circulating biomarkers, diagnosis, prognosis

## Abstract

Melanoma is the most aggressive form of skin cancer, and the majority of cases are associated with chronic or intermittent sun exposure. The incidence of melanoma has grown exponentially over the last 50 years, especially in populations of fairer skin, at lower altitudes and in geriatric populations. The gold standard for diagnosis of melanoma is performing an excisional biopsy with full resection or an incisional tissue biopsy. However, due to their invasiveness, conventional biopsy techniques are not suitable for continuous disease monitoring. Utilization of liquid biopsy techniques represent substantial promise in early detection of melanoma. Through this procedure, tumor-specific components shed into circulation can be analyzed for not only diagnosis but also treatment selection and risk assessment. Additionally, liquid biopsy is significantly less invasive than tissue biopsy and offers a novel way to monitor the treatment response and disease relapse, predicting metastasis.

## 1 Introduction

Over the last 50 years, the incidence of melanoma has grown exponentially, especially in the elderly and populations of fairer skin, those living at lower altitudes (Matthews et al., 2017). Melanoma accounts for more deaths than any other form of skin cancer and commonly metastasizes to other areas of the body (Matthews et al., 2017). Currently, the gold standard for diagnosis of melanoma is performing an excisional biopsy with full resection or an incisional tissue biopsy if the margins are too large. This is an invasive screening with standard risks of localized skin infections. Despite being the current standard of care, only about 20% of suspected melanoma cases are confirmed histologically following excision from dermatologic screening (Carli et al., 2003). Malignant amelanotic lesions typically take an additional 8 years for detection and diagnosis as compared to pigmented malignant melanomas, likely due to the lack of classical risk factors and presentation (Strazzulla et al., 2019). Innovation of diagnostic procedure is necessary to improve mortality rate as the prognosis for survival depends largely on diagnosis before melanoma metastasizes (Millet et al., 2017; Strazzulla et al., 2019).

Utilization of liquid biopsy techniques represent substantial promise in early detection of cancers (Nonaka and Wong, 2022; Nonaka and Wong, 2023). Through this procedure, tumor-specific components shed into circulation are analyzed for both tissue analysis and risk assessment, including recurrence of tumor development and treatment response (Nonaka and Wong, 2018). Additionally, liquid biopsy is significantly less invasive, only requiring peripheral venous access rather than a traditional tissue biopsy, and offers an innovative and novel way to monitor the growth of tumor and response to treatment.

As an adjuvant to traditional biopsies, liquid biopsy has gathered much attention from researchers with over 9,000 published articles and 3,000 registered clinical trials (Chen et al., 2020). We recap current knowledge on the various molecular markers that are currently being researched including: circulating tumor cells (CTCs), circulating tumor DNA (ctDNA), circulation microRNAs (miRNAs), and extracellular vesicles (exosomes). CTCs are highly associated with metastasis, while miRNAs are present in a stable form due to protection form vesicles. In addition to detection of CTCs, ctDNA, and miRNAs, epigenetic alternations, which will be discussed later in this review, can complement the current isolated methods to achieve better performance.

This review summarizes the potential of using liquid biopsy procedure in screening and monitoring the disease progression of melanoma and discusses potential circulating biomarkers for use in diagnosis and monitoring of melanoma progression and treatment response.

## 2 Liquid biopsy in melanoma

**FIGURE 1:**
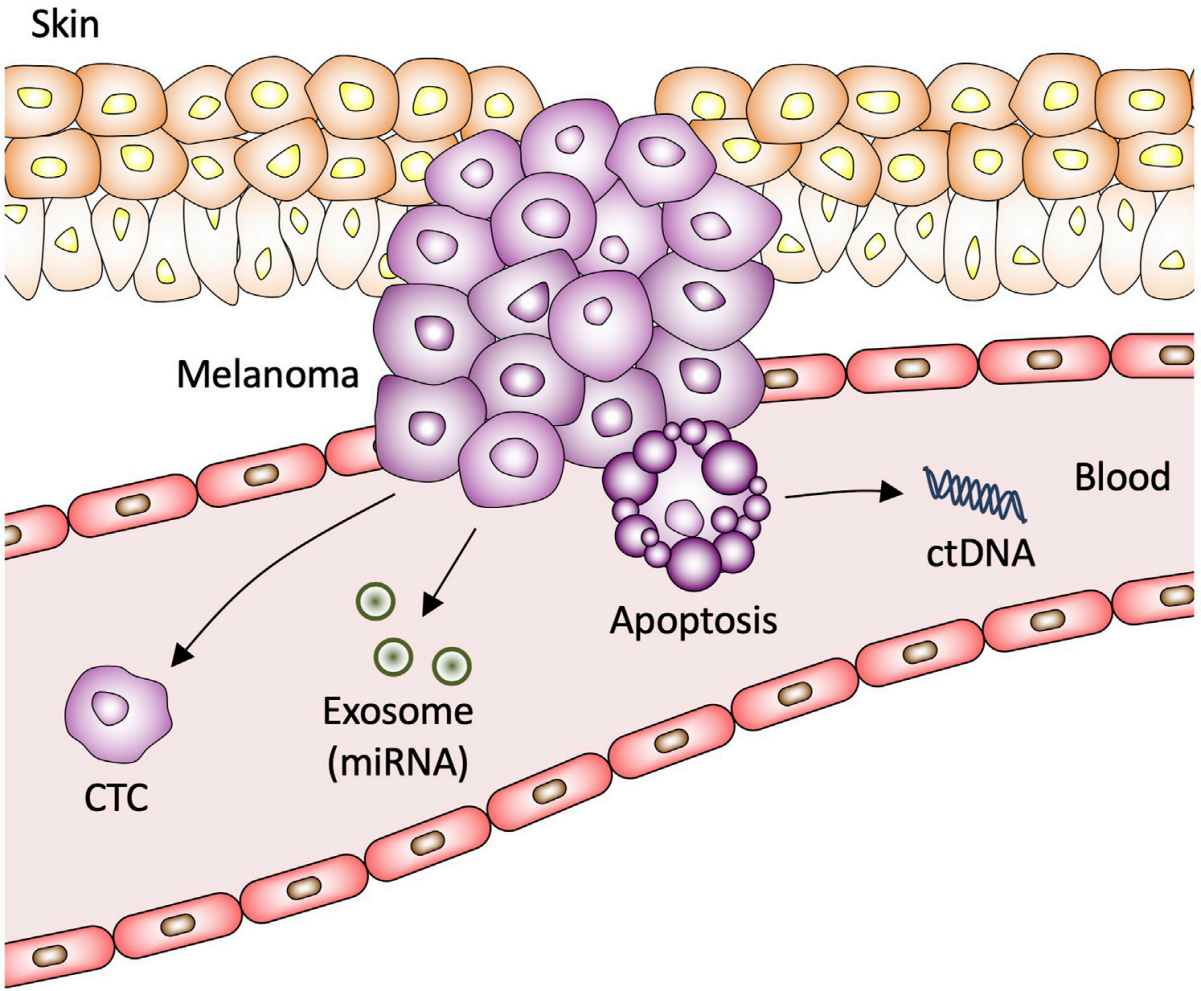
Circulating biomarkers in melanoma. CTC, circulating tumor cell; miRNA, microRNA; ctDNA, circulating tumor DNA.

Over the course of the last decade, several clinical studies have shown promising results with analyzing blood for tumor particles shed into circulation (Alix-Panabières and Pantel, 2021). Originally centered around CTCs, liquid biopsy has quickly expanded to include ctDNAs, proteins, miRNAs, and exosomes (Alix-Panabières and Pantel, 2021). One paramount potential advantage of liquid biopsies is their capability to provide a dynamic picture of the nature of the tumor (Figure 1). Analysis of circulating tumor derivates provides a random sample of the genetic heterogeneity of the tumor, whereas tissue biopsies are constrained by the accessibly of the tumor and only provide insight of tumor nature at the time of collection (Mattox et al., 2019).

### 2.1 Early detection of melanoma by liquid biopsy

**FIGURE 2:**
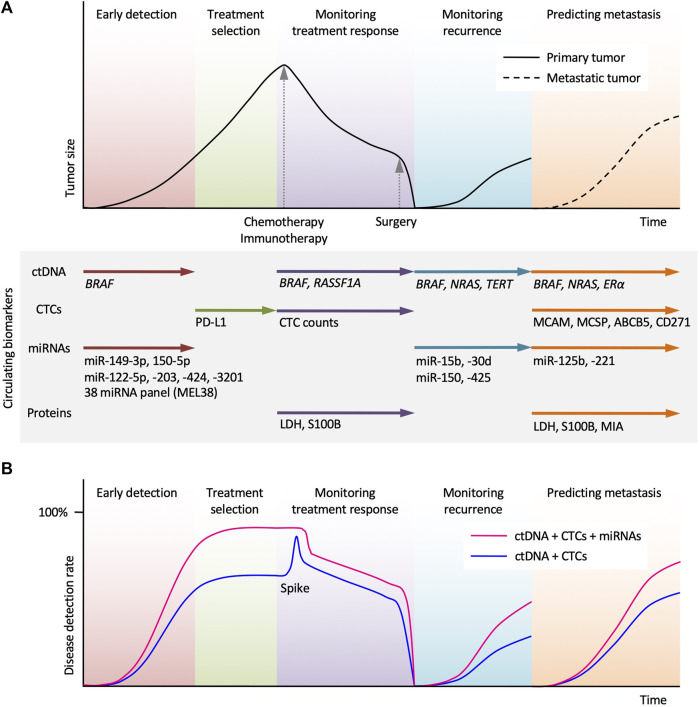
Potential clinical applications of circulating biomarkers in the treatment of melanoma. **(A)** Schematic time course of disease management and tumor size in melanoma patients undergoing chemotherapy (or immunotherapy) and surgery. Circulating tumor DNA (ctDNA) analysis allows early detection, monitoring treatment response, monitoring recurrence, and predicting metastasis. Circulating tumor cells (CTCs) analysis can assist the selection of immune checkpoint inhibitor. miRNAs and protein biomarkers analysis can provide complementary information and help in melanoma diagnosis and treatment. **(B)** Use of miRNAs in combination with ctDNA and CTCs allows higher disease detection. A spike in ctDNA level reflects transient tumor cell death by systemic therapy.

One of the most important prognostic factors for the effective treatment and management of melanoma is early diagnosis. Unfortunately, melanoma is commonly diagnosed in later stages or after metastasis has occurred (Mohammadpour et al., 2019). Current data suggests that inventorying *BRAF* ctDNA is a viable method for diagnosis of melanoma and determination of its stage (Figure 2A) (Kamińska et al., 2021). ctDNA is emerging as one of the most promising biomarkers for early detection of melanoma, providing a way to determine the melanoma progression.

### 2.2 Monitoring treatment response in melanoma by liquid biopsy

Liquid biopsy is currently utilized in the management of other cancers, such as colorectal cancer where it aids in treatment decisions and monitoring treatment response. For example, a study conducted by Ye et al. (2022) demonstrated that changes in the ctDNAs profile correlated with vemurafenib, irinotecan and cetuximab (VIC) therapy response and resistance in a cohort of 29 patients with *BRAF* V600E mutated metastatic colorectal cancer. Within this study, participants who reached complete clearance of *BRAF* V600E ctDNA material had a statistically significant progression-free survival (PFS). In addition, researchers were able to isolate and study other ctDNA material, such as the WNT pathway-related *RNF43*, which when predominate demonstrated a favorable prognosis with a response rate as high as 80% and longer PFS after VIC treatment (Elez et al., 2022; Ye et al., 2022). The use of liquid biopsy after a surgical resection of a colorectal tumor with elevated levels of ctDNAs can be indicative for adjuvant therapy, such as chemotherapy (Mattox et al., 2019).

With this method and identification of more markers, melanomas can be monitored more effectively during treatment regiments using *BRAF* ctDNA levels as an indicator for treatment effectiveness and tolerance (Figure 2A). Additionally, the liquid biopsy is quicker than previous methods allowing for closer monitoring and immediate results with obvious reduction in overall complications and metastasis (Cescon et al., 2020).

### 2.3 Monitoring recurrence in melanoma by liquid biopsy

Minimal residual disease (MRD) refers to the small number of cancer cells that remain in the body after treatment. The occult micrometastases can be seen even after successful treatment with apparent resolution of an early stage cancer, which can result in relapse (Pantel and Alix-Panabieres, 2019). Post-treatment follow-up studies using liquid biopsy methods showed detection of ctDNAs and CTCs for multiple types of cancer allowing detection of MRDs earlier than standard clinical imaging (Pantel and Alix-Panabieres, 2019). The purpose of monitoring recurrence is to prevent the conversion of micrometastases to overt metastases.

Although, there is currently no firm evidence that liquid biopsy can detect MRD in melanoma, multiple studies highlight the promising liquid biopsy in monitoring MRD in other solid tumors. Detection of CTCs in high-risk early-stage breast cancer patients 2 years after completion of chemotherapy has been associated with shorter overall survival (OS) and disease-free survival (DFS) (Trapp et al., 2019). Hormone-positive breast cancer patients with positive CTC status 5 years after diagnosis have 13.1-fold higher recurrence rates (Sparano et al., 2018). In 55 early-stage breast cancer patients, detection of ctDNA with known somatic mutations from the primary tumor was associated with increased risks of metastatic relapse and preceded the clinical diagnosis of relapse by a median of 7.9 months (Sparano et al., 2018). In colorectal cancer, the presence of ctDNA following surgical resection has been associated with significantly higher recurrence rates (Tie et al., 2016). One study found that postoperative detection of ctDNA in colorectal cancer patients had an average lead time of 9.4 months when compared to CT-based detection of recurrence (Scholer et al., 2017). Melanoma patients endure high rates of recurrence and progression after surgical intervention and or therapeutic treatment (Lin et al., 2018). Developing liquid biopsy modalities to effectively monitor MRD would allow for earlier detection of disease relapse resulting in better outcomes. Liquid biopsy may also allow for earlier detection of treatment resistance as MRD is thought to contribute to acquired resistance to targeted therapy (Patel et al., 2023).

### 2.4 Predicting prognosis in melanoma by liquid biopsy

CTCs and ctDNAs can be measured at diagnosis to provide prognostic indication in melanoma (Figure 2A). Higher levels of ctDNAs at diagnosis are associated with poor prognosis in melanoma patients (Cescon et al., 2020). Disease prognosis is also inversely related to the number of CTCs in uveal, cutaneous, and mucosal melanoma (Siravegna et al., 2017; Beasley et al., 2022).

## 3 Circulating tumor cells (CTCs) in melanoma

CTCs are cancer cells that shed off primary solid tumors into the blood stream that function as a seeding mechanism for metastasis (Figure 1). CTCs maintain genetic heterogenicity of the primary tumor and therefore represent significant value for usage in diagnosis, patient prognosis, disease modeling, and monitoring tumor response to drug therapy (Yang et al., 2019). One of the challenges to successful characterization of melanoma CTCs is utilizing a sufficiently diverse representation of biomarkers in collection procedure. Prior studies have focused on using CellSearch^TM^ kits that were developed to isolate CTCs that express melanoma cell adhesion molecule (MCAM) from blood samples and detect CTCs by immunostaining with melanoma-associated chondroitin sulphate proteoglycan (MCSP) (Marsavela et al., 2018). A subsequent study found that using MCAM, MCSP, ATP-binding cassette sub-family B member 5 (ABCB5), and CD271 captured a significantly high proportion of CTCs (Figure 2A) (Marsavela et al., 2018). Early efforts to quantify CTCs utilized polymerase chain reaction (PCR) with immuno-capture enrichment of CTCs via the expression of cell surface antigens (Huang and Hoon, 2016). However, melanoma cells express unique antigens including paired box 3 (PAX3), melanoma-associated antigen recognized by T cells 1 (MART-1/Melan-A), melanoma-associated antigen 3 (MAGE-A3), and polypeptide N-acetylgalactosaminyltransferase (GalNAc-T) that are not expressed by leukocytes circulating in peripheral blood and could therefore be used to detect CTCs directly by quantitative reverse transcription PCR (qRT-PCR) without having to perform CTC isolation or enrichment procedures (Huang and Hoon, 2016). Other common markers used to identify melanoma CTCs include microphthalmia-associated transcription factor (MITF) (Reynolds et al., 2003; Xi et al., 2007; Samija et al., 2010).

### 3.1 Clinical utility of circulating tumor cells (CTCs) as biomarkers in melanoma

One of the major limitations for the clinical utility of CTCs as a liquid biopsy target is their significantly low abundance in peripheral circulation; 1–3 CTCs typically corresponds to 5 billion blood cells. Another point of concern is that many of the antigenic markers expressed by CTCs used for isolation techniques including MCAM, MAGE-A3, MART-1, and HMW-MAA are expressed by benign melanocytes, spermatocytes, other forms of cancerous cells and endothelial cells (Kamińska et al., 2021). Current data suggests that inventorying CTCs is a viable method for diagnosis of melanoma and determination of its stage (Kamińska et al., 2021). CTCs could potentially provide a way to determine the melanoma progression. A study using immunomagnetic enrichment techniques to capture CTCs by utilization of MCAM, MCSP, ABCB5, and CD271 to compare populations of CTCs in circulation between individuals with primary and metastatic melanomas, found that CTC populations were significantly higher in metastatic melanoma (Figure 2A) (Freeman et al., 2012). This suggests that measurement of CTCs could serve as an indicator to melanoma prognosis and a diagnostic tool for metastasis. A study also demonstrated that the volume of CTCs was correlated with the aggressiveness and stage of disease and applied to the overall survival of patients with metastatic melanoma (Ulmer et al., 2004). The levels of MCAM, MAGE-A3, tyrosinase, and melanoma-associated tumor antigen p97 mRNA markers found in CTCs have also been suggested to be representative of disease relapse (Hoon et al., 2000). Research demonstrated that detection of CTCs was significantly associated with disease relapse within 6 months of baseline measurement in newly diagnosed patients with stage III melanoma (Lucci et al., 2020).

Monitoring CTC response to therapeutic interventions could be of immense value for treatment selection for metastatic melanoma. Dynamic fluctuations in CTC profiles are representative of response to therapeutic drugs in melanoma. A recent study demonstrated that CTC decreased in response to vemurafenib treatment correlating with increased OS in patients with metastatic melanoma patients (Klinac et al., 2014). This highlights potential for using CTC profiles to monitor response to vemurafenib treatment. Liquid biopsy characterization could also serve use in identifying patients with drug resistant disease. Approximately 50% of patients with metastatic melanoma harbor mutations for the serine/threonine-protein kinase *BRAF* resulting in constitutive activation of the mitogen activated protein kinase cascade; a significant proportion of these melanomas are intrinsically resistant to BRAF inhibitors while others acquire resistance within a few months (Welsh et al., 2016). CTC populations decreased in patients after receiving immunotherapy for *BRAF*, *NRAS*, and *KIT* mutant alleles in metastatic melanoma, highlighting the utility of monitoring CTC response to treatment to identify *BRAF*-mutant non-responders (Hong et al., 2018). Importantly, PD‐L1 expression on CTCs is predictive of response to pembrolizumab in advanced melanoma (Figure 2A) (Khattak et al., 2020).

Immune checkpoint inhibitors (ICI) have revolutionized treatment for advanced melanoma, as evidenced by impressive rates of tumor regression and survival benefit compared to conventional treatments (Carlino et al., 2021). Data from a recent meta-analysis suggests that for all forms of cancers, PD-L1-positive CTCs were associated with a worse prognosis and shorter PFS and OS (Kong et al., 2021). However, the prescence of PD-L1-positive CTCs in melanoma has been associated with a strong response to ICI. In a study of 40 patients with advanced melanoma undergoing pembrolizumab treatment, patients with detectable PD-L1-positive CTCs had a significantly longer PFS compared with PD-L1-negative patients, suggesting that PD-L1 expression on CTCs may predict response to pembrolizumab (Khattak et al., 2020). This data suggests that analyzing patients’ individualized CTC PD-L1 expression profile may provide substantial benefit in the clinical decision-making process and allow for personalized treatment based on liquid biopsy results (Figure 2A).

### 3.2 Clinical trials for CTCs in melanoma

Currently, there are five clinical trials related to the CTCs in melanoma (Table 1). Table 1 shows several studies that are focused on the treatment response and how CTCs can play a role in prognosis, while others are focused on the survival evaluation of patients with melanoma or metastatic melanoma. Interestingly, there is one study focused on using heat shock protein (HSP) 70 to better characterize CTCs due to their low abundance in blood samples and compare those results to current methods of measuring CTCs. There is a decrease in the number of active, recruiting, and completed trials when compared to previous papers that have summarized clinical trial findings; however, the trials are more diverse in the nature of their outcomes than before (Boyer et al., 2020).

**TABLE 1:** Clinical trials on CTCs in melanoma listed on ClinicalTrials.gov database. No results were posted to ClinicalTrials.gov for all clinical trials listed here as of June 2024.

ClinicalTrials ID	Year	Location	Enrollment	Objective	Status	Ref
NCT01573494	2012	France	30	Detect CTCs in melanoma	Completed	NA
NCT01558349	2012	France	73	Compare EPISPOT and CellSearch	Completed	NA
NCT01528774	2012	United States	150	Characterize CTCs in melanoma	Completed	NA
NCT03797053	2019	France	450	Develop CTC marker for predicting disease progression	Unknown	NA
NCT04628806	2020	Germany	120	Compare HSP70 and EpCAM for CTC isolation	Unknown	NA

NA, not applicable.

## 4 Circulating tumor DNA (ctDNA) in melanoma

ctDNA is highly fragmented double-stranded DNA that circulates in blood, saliva, urine, and cerebrospinal fluid (Sacco et al., 2020; Nonaka and Wong, 2022). Increase in cell-free DNA (cfDNA) is observed in several pathological conditions including cardiovascular disease, infections, heart failure, and cancer (Thierry et al., 2016). ctDNA is DNA released into circulation by necrotic or apoptotic cells of the primary tumor (Figure 1) (Alix-Panabières et al., 2012). ctDNA is typically released in early developmental stages of tumor growth and commonly travel in circulation as nucleosomes, indicating that ctDNA possesses original features of nuclear chromatin for the tumor (Alix-Panabières et al., 2012). Solid tumor’s ctDNA is primarily released into circulation after necrosis; it has been well established that ctDNA levels are higher in patients with advanced cancer compared to low-grade cancer (Marzese et al., 2013). Like other liquid biopsy methods, one of the major challenges for using ctDNA as a clinically viable blood-based biomarker is developing highly sensitive methods to detect ctDNA. Dilution of tumor-derived DNA with normal DNA in circulation requires methodology that can detect DNA variants at low mutant allelic frequencies in the cfDNA; current methods for detection of ctDNAs include qPCR, emulsion-PCR and next-generation sequencing (NGS) (Sacco et al., 2020). A major concern with ctDNA analysis as a method of routine diagnostic procedure is that melanoma is a heterogenous tumor typically composed of several subclones (Yancovitz et al., 2012). A source of concern is that ctDNA harboring specific genetic mutations can theoretically originates from any subclone rather than the predominant clone or from the premalignant lesion (Gaiser et al., 2018).

### 4.1 Clinical utility of circulating tumor DNA (ctDNA) as biomarkers in melanoma

Analysis of ctDNA for tumor-specific mutations is a promising application of liquid biopsy technique for determination of prognosis in cancers. Tumor volume, location and cellular turnover influence the levels of ctDNA in melanoma patients (Jahr et al., 2001; Heitzer et al., 2015). ctDNA levels in melanoma are associated with tumor burden; a past study demonstrated that ctDNA levels correlated significantly with other indicators of disease burden in melanoma patients including lactate dehydrogenase (LDH) and melanoma inhibitory activity (MIA) (Sanmamed et al., 2015). A recent study found that *BRAF* mutations in ctDNA are detectable in more than 75% of melanoma patients harboring *BRAF* V600E/V600K-positive tumors, suggesting that lacking *BRAF* mutation-positive ctDNA may be a prognostic indicator for better outcome for metastatic melanoma patients (Santiago-Walker et al., 2016). Patients negative for *BRAF* mutation-positive ctDNA also had better responses to MEK inhibitor (trametinib) and BRAF inhibitor (dabrafenib) (Santiago-Walker et al., 2016). Low baseline ctDNA levels are associated with long-term clinical benefits and can be useful in predicting patient response to immunotherapy (Gray et al., 2015). A phase II clinical trial demonstrated that high baseline levels of ctDNA were correlated with a lower response rate and PFS in targeted therapeutic interventions with the BRAF inhibitor dabrafenib (Ascierto et al., 2013). It has also been reported in study comparing LDH to ctDNA levels, that elevated ctDNA levels had higher sensitivity than LDH for determining disease progression (Chang et al., 2016). *BRAF*, *NRAS* and *TERT* mutations are present in more than 66% of melanomas; a study performed in 2020 measuring ctDNA levels harboring these mutations in 19 different patients with late-stage melanoma concluded that detectable levels of ctDNA had a shorter PFS and shorter median time for disease progression at 50 days compared to 146 days for patients that did not have detectable ctDNA (Figure 2A) (Marczynski et al., 2020).

Quantification of ctDNA has also been shown to be useful indicator of response to treatment. ctDNA harboring *BRAF* V600E mutation has shown promise to monitor treatment response to BRAF inhibitors (Figure 2A). Higher *BRAF* V600E ctDNA concentrations correlate to disease progression and initial tumor burden while lower concentrations are associated with initial treatment response and higher PFS and OS (Sanmamed et al., 2015). Programmed cell death protein 1 (PD-1) inhibitors are foundational therapeutics in the treatment of metastatic melanoma, and assessment of *BRAF* V600E/V600K ctDNA levels in patients receiving anti-PD-1 treatment has been shown to be an accurate predictive factor for tumor response, PFS, and OS (Lee et al., 2017). It has also been demonstrated that levels of mutant *BRAF* and *NRAS* ctDNA concentrations in patients with metastatic melanoma correlated with decreased metabolic activity monitored by fluorodeoxyglucose positron emission tomography (FDG-PET) in response to therapeutic interventions (Wong et al., 2017). ctDNA analysis is also effective in the identification of acquired resistance mutations. *NRAS* mutations have been identified in 8%–26% of patients with acquired BRAF inhibitor resistance. ctDNA collected from patients that had initially responded to BRAF inhibitors (dabrafenib/trametinib) harbored *NRAS* mutations following development of treatment resistance that where not present prior to treatment initiation (Gray et al., 2015).

*KRAS* mutations promote and maintain tumor growth through the RAS/MAPK pathway and are thought to be present in approximately 25% of all human cancers (Mustachio et al., 2021). *KRAS* mutations are associated with a poor prognosis in melanoma patients (Zhou et al., 2024). There is a high concordance level in *KRAS* mutations between ctDNA and primary tumor tissue (Bettegowda et al., 2014). Analysis of ctDNA during EGFR inhibitor therapy has shown rapid emergence of *KRAS* and *NRAS* mutations due to selective pressure; withdrawal of EGFR inhibitory therapy results in a decrease in *KRAS*-mutant subclones (Siravegna et al., 2015). This suggests that mutant ctDNA reflects dynamic changes in the tumor microenvironment that can be utilized to direct clinical decision making in timing and selection of target therapeutics. Upregulation of EGFR has also been associated with BRAF inhibitor resistance, indicating a potential role for ctDNA in early identification of resistance to vemurafenib and dabrafenib in metastatic melanoma (Ji et al., 2021). Characterization of *EGFR* mutations has been a specific focus of ctDNA liquid biopsy as they have demonstrated high specificity for specific mutations used to guide therapeutic interventions in patients with lung cancer (Nakamura et al., 2011). Liquid biopsy with ctDNA may play a similar role in melanoma as increasing evidence suggests that EGFR overactivity is involved in tumorigenesis and resistance mechanisms (Simiczyjew et al., 2019; Pastwinska et al., 2022).

### 4.2 Epigenetic-based liquid biopsy

In addition to the abnormal genetic changes in ctDNA due to the oncogenic process, epigenetic changes represent another avenue of utility that could potentially be used in conjunction with ctDNA mutations to provide better accuracy for these test (Chen et al., 2020). For example, a review by Li et al. (2022) showed that ctDNA methylation occurs earlier in malignant lung cancers and can be a potent biomarker to aid ctDNA sequencing. Epigenetic alterations in ctDNA have also been shown as potential prognostic factors. Several genes methylated in primary and metastatic melanomas were detected in serum as methylated circulating DNA (Hoon et al., 2004). Circulating methylated *RASSF1A* DNA was more commonly found in melanoma patients that were nonresponsive to chemotherapy (Figure 2A) (Mori et al., 2006). Circulating methylated *ESR1* encoding Estrogen Receptor alpha (ER-α) was also correlated with worse prognosis in melanoma patients (Figure 2A) (Mori et al., 2006). Since epigenetic profiles vary between cases, it may be challenging to create a sensitive and specific ctDNA methylation profile. However, epigenetic alterations can differ between cell types, covering the shortcomings of ctDNA mutation profiling alone, with the most extensively studied being ctDNA methylation (Chen et al., 2020).

### 4.3 Clinical trials for ctDNA in melanoma

There are currently eleven active, recruiting, completed, terminated, or unknown clinical trials that are using ctDNA in some capacity with relation to melanoma (Table 2). Ten of the eleven trials are focusing on prognostic studies that associate the levels of ctDNA in patients with melanoma to PFS or OS (Table 2). Three of these trials are focusing on the pre- and post-treatment outcomes of various therapies.

**TABLE 2:** Clinical trials on ctDNA in melanoma listed on ClinicalTrials.gov database. No results were posted to ClinicalTrials.gov for all clinical trials listed here as of June 2024.

ClinicalTrials ID	Year	Location	Enrollment	Objective	Status	Ref
NCT01334008	2011	France	40	Develop ctDNA detection technique in choroidal melanoma	Completed	NA
NCT02251314	2014	Canada	12	Assess tumor heterogeneity in *BRAF*-mutant melanoma	Completed	NA
NCT02849145	2016	France	60	Evaluate ctDNA in metastatic uveal melanoma	Completed	NA
NCT03416933	2018	France	35	Monitor TKIs treatment response in *BRAF*-mutant melanoma	Completed	NA
NCT03517332	2018	United States	10,000^a^	Detect mutations in ctDNA in solid tumors	Unknown	NA
NCT03808441	2019	United Kingdom	21	Monitor tumor burden in *BRAF*-mutant melanoma	Active, not recruiting	NA
NCT04354064	2020	United States	100	Assess treatment response in solid tumors	Recruiting	NA
NCT04866680	2021	France	165	Predict resistance to immunotherapy in melanoma	Recruiting	NA
NCT04901988	2021	United Kingdom	8	Assess response to immunotherapy in melanoma	Terminated^b^	NA
NCT04761783	2021	United States	1,539	Assess response to immunotherapy in solid tumors	Active, not recruiting	Kasi et al. (2022)
NCT05196087	2022	Canada	500	Detect molecular residual disease before metastasis	Recruiting	NA

^a^ Prospective, multi-center, blinded feasibility study in different types of solid tumors.

^b^ Terminated: Closed earlier than expected due to the need for a redesign to reflect the recent change in standard of care guidelines. NA, not applicable.

## 5 Other circulating biomarkers in melanoma

### 5.1 Proteins

Currently lactate dehydrogenase (LDH) is the only serum protein that is used in melanoma-staging procedure per the most recent edition of the AJCC Cancer Staging Manual (Gershenwald et al., 2017). LDH is thought to be released into circulation with melanoma cells outgrow their vascular supply as it is responsible for the conversion of pyruvate to lactate in low oxygen environments (Balch et al., 2001). In a study conducted with 1,085 late-stage melanoma patients, elevated LDH concentration correlated with worsened survival rates but interestingly did not correlate with tumor size or metastatic sites (Agarwala et al., 2009). Serum LDH levels have also shown promise in monitoring disease response and predicting PFS and OS rates in patients receiving dabrafenib and trametinib treatment for metastatic BRAF-mutant melanoma (Table 3) (Long et al., 2016). Elevated LDH levels are a poor prognostic metric for anti-CTLA-4 treatment (ipilimumab) for metastatic melanoma and indicative of poor OS (Figure 2A) (Diem et al., 2015).

**TABLE 3:** Protein biomarkers with prognostic outcomes as well as specificity and sensitivity for melanoma.

Marker	Level	PFS (months)	OS (months)	Specificity (%)	Sensitivity (%)	Ref
LDH	Normal	17.4	45.5	N/A	N/A	Long et al. (2016)
	1 ≤ ULN < 2	7.4	17.0	N/A	N/A	
	2 ≤ ULN	5.5	8.8	100	42.9	
S100B	Normal	38.4	94.8	N/A	N/A	Tarhini et al. (2009)
	Elevated (≥0.15 g/L)	16.8	55.2	50.0	58.3	
MIA	Normal	24	N/A	N/A	N/A	Sanmamed et al. (2014)
	Elevated (≥9.0 μg/L)	4	N/A	100	81.8	

PFS, progression-free survival; OS, overall survival; N/A, not applicable; ULN, upper limit normal.

Members of the S100 protein family are known to play different roles, including influencing cytoskeletal structure, macrophage activation, modulation of cell proliferation, inflammation, and carcinogenesis (Salama et al., 2008). S100B is commonly expressed by melanoma and is known to directly interact with p53 tumor suppressor protein, promoting tumorigenesis in melanoma (Lin et al., 2010). S100B is currently one of the best characterized biomarkers of melanoma and has been routinely used as an immunohistochemical marker for diagnosis of malignant melanoma (Palmer et al., 2011). Increasing levels of S100B correlate with disease progression, and levels of S100B are significantly higher in patients with stage IV melanoma than individuals with early-stage disease (Palmer et al., 2011). Studies have also shown that S100B levels are dynamic representations of disease status and fluctuate as the disease progresses and regresses, suggesting that monitoring S100B could be a valuable metric in monitoring treatment response (Figure 2A) (Hamberg et al., 2003). Elevated baseline S100B levels are also associated with decreased OS in late-stage melanoma patients receiving anti-PD-1 and anti-CTLA-4 therapeutic interventions (Felix et al., 2016; Wagner et al., 2018). Serum S100B levels show great potential as a prognostic marker as well (Tarhini et al., 2009), and several reports indicate that increasing S100B levels correlate with aggressive disease and reduced survival (Figure 2A) (von Schoultz et al., 1996; Abraha et al., 1997; Hauschild et al., 1999; Karnell et al., 1997; Bonfrer et al., 1998).

Melanoma inhibitory activity (MIA) protein is highly expressed by melanoma cells but not by normal melanocytes (Bosserhoff, 2005). *In vivo*, MIA inhibits melanoma cell attachment to laminin/fibronectin via blocking of integrin binding sites allowing for invasion and metastasis (Bosserhoff et al., 2003). A study quantified levels of MIA in serum using ELISA in comparison to S100 and soluble intercellular adhesion molecule 1 (ICAM-1) and suggested that MIA was the more sensitive biomarker and found that enhanced MIA serum levels where present in 100% of patients with stage III or IV melanoma (Bosserhoff et al., 1997). MIA represents a promising serum marker for systemic malignant melanoma with high sensitivity and specificity (Sanmamed et al., 2014). Since this study was conducted, recent literature suggests that higher MIA levels are associated with short OS and disease-free survival (DFS) and that monitoring S100B, MIA, and LDH serum levels could prove useful in prediction of OS and DFS (Figure 2A) (Schultz et al., 1998; Garbe et al., 2007; Garbe et al., 2008; Nikolin et al., 2016).

### 5.2 microRNAs (miRNAs)

miRNAs are single-stranded molecules of noncoding RNAs that average 20–24 nucleotides in length that negatively regulate gene expression to influence cellular differentiation, proliferation, and apoptosis (Esquela-Kerscher and Slack, 2006). miRNAs influence both the development and progression of cancer (Calin and Croce, 2006; Esquela-Kerscher and Slack, 2006). Expression of miRNAs is characteristically dysregulated in malignancy by epigenetic alterations, chromosomal abnormalities, and changes in transcriptional control mechanisms (Chen et al., 2012).

One of the most appealing characteristics of using miRNAs as a blood-based biomarker for liquid biopsy techniques is their stability. miRNAs in circulation are protected from endogenous RNase activity by exosome packaging before secretion from the melanoma cells (Mitchell et al., 2008). Commonly used methods of detection for miRNAs include qRT-PCR and microarray; new techniques including rolling circle amplification (RCA) and loop-mediated isothermal amplification (LAMP) have also been developed to detect serum miRNAs more efficiently (Ye et al., 2019).

Several different miRNAs have been studied in correlation with melanoma (Table 4). For example, metastatic melanoma patients have higher levels of miR-221 than healthy controls (Kanemaru et al., 2011; Li et al., 2014). miR-221 levels are increased with stage I-IV melanoma compared to melanoma *in situ* and miR-221 correlated with the thickness of the tumor (Kanemaru et al., 2011). Higher serum miR-221 levels are also associated with a worse OS rate for cutaneous malignant melanoma (Figure 2A) (Li et al., 2014). Additionally, miR-122-5p, miR-203, miR-424, and miR-3201 have been detected in the sera of patients with melanoma (Figure 2A) (Margue et al., 2015; Sahranavardfard et al., 2019).

**TABLE 4:** Summary of potential miRNA markers in liquid biopsy and their primary diagnostic and prognostic associations in melanoma.

miRNA marker	Expression	Clinical utility	Ref
miR-125b	Upregulated	Diagnostic for uveal melanoma metastases	Achberger et al. (2014)
miR-221	Upregulated	Prognostic for stage, worse PFS and OS	Kanemaru et al. (2011), Li et al. (2014)
miR-149-3p, miR-150-5p	Upregulated	Diagnostic for melanoma patients (stage I–IV)	Fogli et al. (2017)
miR-15b, miR-30d, miR-150, miR-425	Upregulated	Prognostic for disease recurrence	Fleming et al. (2015)
miR-122-5p, miR-203, miR-424, miR-3201	Upregulated	Diagnostic for melanoma patients	Margue et al. (2015), Sahranavardfard et al. (2019)
38 miRNAs panel (MEL38)^a^	Upregulated	Distinguish stages with high sensitivity and specificity	Van Laar et al. (2018), Van Laar et al. (2023)
miR-16	Downregulated	Diagnostic for early and late-stage melanoma	Guo et al. (2016)
miR-193a-3p	Downregulated	Diagnostic for melanoma patients (stage I–IV)	Fogli et al. (2017)
miR-206	Downregulated	Prognostic for worse PFS and OS	Tian et al. (2015)
miR-429	Downregulated	Diagnostic for lymphatic metastases	Sun et al. (2021)
miR-29c-5p, miR-324-3p	Downregulated	Distinguish melanoma from renal and colon cancer	Greenberg et al. (2013)

^a^ 38 miRNAs panel comprises miR-424-5p, miR-548l, miR-34a-5p, miR-497-5p, miR-299-3p, miR-205-5p, miR-1269a, miR-624-3p, miR-138-5p, miR-1-5p, miR-152-3p, miR-1910-5p, miR-181b-5p, miR-3928-3p, miR-3131, miR-301a-3p, miR-1973, miR-520d-3p, miR-548a-5p, miR-548ad-3p, miR-454-3p, miR-4532, miR-1537-3p, miR-553, miR-764, miR-1302, miR-1258, miR-522-3p, miR-1264, miR-1306-5p, miR-219a-2-3p, miR-431-5p, miR-450a-5p, miR-2682-5p, miR-337-5p, miR-27a-3p, miR-4787-3p, and miR-154-5p.

PFS, progression-free survival; OS, overall survival.

The literature highlighting the potential of miRNAs as useful biomarkers for melanoma treatment is impressive. For example, microarray profiling performed on plasma samples from 32 patients with stage I-IV melanoma and 16 healthy controls lead to the identification of a 38-miRNA signature (MEL38) that had the ability to distinguish between melanoma patients of varying stages and healthy controls with both high specificity and sensitivity (Figure 2A) (Van Laar et al., 2018; Van Laar et al., 2023). miR-29c-5p and miR-324-3p levels are markedly lower in the serum of metastatic melanoma patients compared to healthy controls and can be used to distinguish melanoma for renal and colon cancer (Greenberg et al., 2013). Upregulation of miR-149-3p and miR-150-5p and downregulation of miR-193a-3p has been demonstrated to discriminate melanoma patients (stage I-IV) from healthy individuals at a high capacity (Figure 2A) (Fogli et al., 2017). Plasma levels of miR-125b are elevated in uveal melanoma patients compared to health controls; miR-125b levels are also more elevated at metastasis compared to the levels at the initial diagnosis (Achberger et al., 2014).

Serum levels of miR-15b, miR-30d, miR-150, and miR-425 have been used to stratify patients into risk groups for disease recurrence with high sensitivity (Figure 2A) (Fleming et al., 2015). Decreases in miR-206 serum levels are associated with poor prognosis and advanced disease progression (Tian et al., 2015). Reports also suggest that expression of miR-221 correlates to traditional pathologic melanoma prognostic characteristics including tumor thickness, higher T and N classification, poor differentiation, metastasis, and advanced clinical stage (Li et al., 2014). Higher serum levels of miR-221 are also associated with a significantly lower recurrence-free survival rate and 5-year survival rate (Li et al., 2014). Interestingly, circular RNA (circRNA_0082835) was demonstrated to promote lymphatic metastasis of melanoma by inhibition of miR-429 (Sun et al., 2021). The study also concluded that levels of miR-429 decreased with lymphatic metastasis, suggesting the potential value of miR-429 for monitoring melanoma disease progression (Sun et al., 2021).

A major hurdle to the implementation of miRNA as biomarker in the treatment of melanoma is developing sufficient collection and amplification procedure for reliable analysis. For example, miR-16 was reported to be downregulated in the serum of early-stage melanoma patients (stage I-II) compared to health controls and is markedly downregulated in the serum of late-stage melanoma patients (stage III-IV) compared to early-stage patients (Guo et al., 2016). Conversely another study using a different miRNA isolation kit found that miR-16 levels increased in the serum of melanoma patients compared to healthy controls (Stark et al., 2015). Conflicting results between these two studies could potentially be attributed to variation in the sensitivity and specificity of different qRT-PCR assays paired with variations in miRNA extraction kits.

### 5.3 Extracellular vesicles (exosomes)

Exosomes are a subset of extracellular vesicles that originate from vastly diverse cell populations (Yáñez-Mó et al., 2015). The lipid membranes of exosomes provide a protective function by protecting the exosome contents from degradation from physiological enzymes found in body fluids, including blood, saliva, urine, and cerebrospinal fluid (Yáñez-Mó et al., 2015; Nonaka and Wong, 2017). Exosomes contain several differing populations of physiologically significant molecules, including DNA, messenger RNAs (mRNAs), miRNAs, and proteins (Wang et al., 2016; Cheng et al., 2019). Exosomes play a critical role in maintaining the tumor microenvironment, transporting proteins, modulating signaling pathways, drug resistance, and metastasis (Webber et al., 2010; Peinado et al., 2012). Exosomes are secreted by cells, traverse the lymphatic system, and then enter circulation (Figure 1) (Caby et al., 2005).

Exosomes derived from melanoma cells contain key differences that allow for discrimination from normal melanocytes (Xiao et al., 2012). A study found that MIA and S100B proteins could be isolated from melanoma-derived exosomes at concentrations that significantly correlated with serum concentrations (Table 5) (Alegre et al., 2016). Exosomal MIA and S100B levels were also higher in late-stage melanoma patients and associated with shorter survival (Alegre et al., 2016).

**TABLE 5:** Summary of potential exosomal markers in liquid biopsy with relation to the overall survival in patients with melanoma.

Exosomal marker	Level	AUC	OS (months)	Ref
S100B	Normal (<0.03 μg/L)	0.840	10	Alegre et al. (2016)
	Elevated (≥0.03 μg/L)		7	
MIA	Normal (<2.5 μg/L)	0.883	11	Alegre et al. (2016)
	Elevated (≥2.5 μg/L)		4	

AUC, area under the curve.

Characterization of exosomes from melanoma cells shows significant potential for predicting and monitoring response to therapy. Metastatic melanoma cells release high levels of exosomes that carry PD-L1 on their surface; IFN-g facilitates upregulation of exosomal PD-L1, promoting tumor growth by inhibition of CD8^+^ T cells (Chen et al., 2018). Characterization of PD-L1 levels in exosomes shows promise in predicting a patient’s response to ICI. A prior study analyzing the amount of exosomal PD-L1 in stage III-IV melanoma patients before and after treatment with pembrolizumab found that higher exosomal PD-L1 levels were associated with worse clinical outcomes as T cells cannot overcome the solid inhibitory effect (Chen et al., 2018). Similarly, another study found that high PD-L1 levels and low exosomal CD28 expression negatively impact PFS in patients undergoing anti-PD-1 immunotherapy (Zhang et al., 2020). This suggests that higher pretreatment exosomal PD-L1 levels are likely to make immunotherapy ineffective due to diminished CD8^+^ T cell response. Reductions in exosomal PD-L1 during ICI have also been shown to correlate with radiographic evidence of tumor response on PET-CT and CT scans, suggesting that exosomal expression of PD-L1 is directly related to a patient’s response to ICI therapy (Cordonnier et al., 2020).

Cancer cells communicate with their microenvironment through exosomes and microvesicles to facilitate tumor growth, metastasis, immune system evasion, and angiogenesis. Exosomes play a part in complex cellular interactions between tumor cells and the tumor microenvironment at every step of cancer progression, therefore, characterization of exosomes provides real-time information about how tumor cells interact with surrounding tissues (Hu et al., 2022; Wang et al., 2023). Exosomes alter cells in the tumor microenvironment leading to immunosuppressive effects and decreased response to immunotherapy (Morrissey et al., 2021). The complex interaction between exosomes and the tumor microenvironment makes determining the accuracy of liquid biopsy testing a significant challenge. Tumor immunopathology is incredibly complicated and is incompletely understood (Ilie et al., 2017). Additionally, microenvironmental factors have been shown to influence the release of materials used for liquid biopsies further complicating the accuracy of liquid biopsy (Raimondi et al., 2017).

## 6 Conclusions and future perspectives

While incisional and excisional biopsies remain gold standard for melanoma detection, its limitations can be overcome with alternative methods. Liquid biopsy can provide a much broader analysis of melanoma while remaining less invasive than current methodology. This method can not only provide early detection of melanoma but also long-term protection against metastasis using ctDNA or CTCs. This method provides a novel approach against devastating complications of an otherwise very treatable disease. Additionally, liquid biopsy could be repeated several times throughout a treatment regimen to determine response to treatment, resistance, and prognosis of the melanoma (Alix-Panabières and Pantel, 2021).

Recent shifts from CTCs to ctDNA for early detection and monitoring disease have shown promising with more recognition in recent studies. Using ctDNA as a primary marker indicates quicker diagnosis and thus treatment options for patients. Additionally, protein markers can provide high specificity; however, many of these markers have sensitivity limitations that reduce their clinical validity for recurrence detection. MIA can be further investigated as its sensitivity and specificity are high. S100B protein has a short half-life which can be utilized in evaluation of treatment efficacy.

Due to the rapid evolving nature of liquid biopsy, other new technologies, such as artificial intelligence (AI), could be an additional complement to liquid biopsy in the future. AI has been used in the past with magnetic resonance imaging (MRI) and other imaging modalities to diagnose and monitor patients with cancer; therefore, it is only reasonable to assume that its application in liquid biopsy to create detailed spatial and functional assessments of disease and determining risk is the natural progression of these technologies (Foser et al., 2024). In colorectal cancer, AI has shown promising results with genetic testing analysis and liquid biopsy. Due to enormous volume of data that is required by liquid biopsy, AI machine learning can be implemented where other methodologies have failed to sort, analyze, and evaluate with one study, stating that AI achieved clinical sensitivity and specificity of 80% in a CTC preparation and 70% in a serum protein biomarker in colorectal cancer (Ginghina et al., 2022). Liquid biopsy complimented with AI has an extremely promising future. Although the widespread application of liquid biopsy is exciting and promising, there are some technological limitations to the clinical implementation. For clinics to implement liquid biopsy, it would require tremendous time and monetary resources for AI machine learning, new sequencing machines, software, cloud storage required to store tremendous information, training and technicians for wet laboratory work, data analysts, and a board of individuals to assist (Ignatiadis et al., 2021).

From review, clinical validity can be established for both ctDNA and CTCs in melanoma detection; however, future studies should focus on identifying and improving individual tumor markers for better patient stratification and tumor typing. Currently, highly significant levels of select miRNA tumor markers in patients with melanoma could be evidence of superior biomarkers, which should be explored further (Jones and Nonaka, 2024). Use of miRNAs in combination with ctDNA and CTCs would allow higher disease detection (Figure 2B). While more planning and integration into the clinical workflow is needed, the potential of early diagnosis, continuous treatment monitoring, predicting metastasis, and advancements to precision oncological medicine warrant optimistic conclusions.

## References

[B1] Abraha H. D. Fuller L. C. DU Vivier A. W. P. Higgins E. M. Sherwood R. A. (1997). Serum S-100 protein: a potentially useful prognostic marker in cutaneous melanoma. Br. J. Dermatology 137 (3), 381–385. 10.1111/j.1365-2133.1997.tb03742.x 9349333

[B2] Achberger S. Aldrich W. Tubbs R. Crabb J. W. Singh A. D. Triozzi P. L. (2014). Circulating immune cell and microRNA in patients with uveal melanoma developing metastatic disease. Mol. Immunol. 58 (2), 182–186. 10.1016/j.molimm.2013.11.018 24370793 PMC4143188

[B3] Agarwala S. S. Keilholz U. Gilles E. Bedikian A. Y. Wu J. Kay R. (2009). LDH correlation with survival in advanced melanoma from two large, randomised trials (Oblimersen GM301 and EORTC 18951). Eur. J. Cancer 45 (10), 1807–1814. 10.1016/j.ejca.2009.04.016 19419855

[B4] Alegre E. Zubiri L. Perez-Gracia J. L. González-Cao M. Soria L. Martín-Algarra S. (2016). Circulating melanoma exosomes as diagnostic and prognosis biomarkers. Clin. Chim. Acta 454, 28–32. 10.1016/j.cca.2015.12.031 26724367

[B5] Alix-Panabières C. Pantel K. (2021). Liquid biopsy: from discovery to clinical application. Cancer Discov. 11 (4), 858–873. 10.1158/2159-8290.CD-20-1311 33811121

[B6] Alix-Panabières C. Schwarzenbach H. Pantel K. (2012). Circulating tumor cells and circulating tumor DNA. Annu. Rev. Med. 63, 199–215. 10.1146/annurev-med-062310-094219 22053740

[B7] Ascierto P. A. Minor D. Ribas A. Lebbe C. O'Hagan A. Arya N. (2013). Phase II trial (BREAK-2) of the BRAF inhibitor dabrafenib (GSK2118436) in patients with metastatic melanoma. J. Clin. Oncol. 31 (26), 3205–3211. 10.1200/JCO.2013.49.8691 23918947

[B8] Balch C. M. Buzaid A. C. Soong S.-J. Atkins M. B. Cascinelli N. Coit D. G. (2001). Final version of the American joint committee on cancer staging system for cutaneous melanoma. J. Clin. Oncol. 19 (16), 3635–3648. 10.1200/JCO.2001.19.16.3635 11504745

[B9] Beasley A. B. Chen F. K. Isaacs T. W. Gray E. S. (2022). Future perspectives of uveal melanoma blood based biomarkers. Br. J. Cancer 126 (11), 1511–1528. 10.1038/s41416-022-01723-8 35190695 PMC9130512

[B10] Bettegowda C. Sausen M. Leary R. J. Kinde I. Wang Y. Agrawal N. (2014). Detection of circulating tumor DNA in early- and late-stage human malignancies. Sci. Transl. Med. 6 (224), 224ra24. 10.1126/scitranslmed.3007094 PMC4017867 24553385

[B11] Bonfrer J. M. G. Korse C. M. Nieweg O. E. Rankin E. M. (1998). The luminescence immunoassay S-100: a sensitive test to measure circulating S-100B: its prognostic value in malignant melanoma. Br. J. Cancer 77 (12), 2210–2214. 10.1038/bjc.1998.368 9649135 PMC2150414

[B12] Bosserhoff A.-K. (2005). Melanoma inhibitory activity (MIA): an important molecule in melanoma development and progression. Pigment Cell. Res. 18 (6), 411–416. 10.1111/j.1600-0749.2005.00274.x 16280006

[B13] Bosserhoff A. K. Kaufmann M. Kaluza B. Bartke I. Zirngibl H. Hein R. (1997). Melanoma-inhibiting activity, a novel serum marker for progression of malignant melanoma. Cancer Res. 57 (15), 3149–3153. 9242442

[B14] Bosserhoff A. K. Stoll R. Sleeman J. P. Bataille F. Buettner R. Holak T. A. (2003). Active detachment involves inhibition of cell-matrix contacts of malignant melanoma cells by secretion of melanoma inhibitory activity. Lab. Investig. 83 (11), 1583–1594. 10.1097/01.lab.0000097191.12477.5d 14615412

[B15] Boyer M. Cayrefourcq L. Dereure O. Meunier L. Becquart O. Alix-Panabières C. (2020). Clinical relevance of liquid biopsy in melanoma and merkel cell carcinoma. Cancers (Basel) 12 (4), 960. 10.3390/cancers12040960 32295074 PMC7226137

[B16] Caby M.-P. Lankar D. Vincendeau-Scherrer C. Raposo G. Bonnerot C. (2005). Exosomal-like vesicles are present in human blood plasma. Int. Immunol. 17 (7), 879–887. 10.1093/intimm/dxh267 15908444

[B17] Calin G. A. Croce C. M. (2006). MicroRNA signatures in human cancers. Nat. Rev. Cancer 6 (11), 857–866. 10.1038/nrc1997 17060945

[B18] Carli P. Mannone F. De Giorgi V. Nardini P. Chiarugi A. Giannotti B. (2003). The problem of false-positive diagnosis in melanoma screening: the impact of dermoscopy. Melanoma Res. 13 (2), 179–182. 10.1097/00008390-200304000-00011 12690302

[B19] Carlino M. S. Larkin J. Long G. V. (2021). Immune checkpoint inhibitors in melanoma. Lancet. 398 (10304), 1002–1014. 10.1016/S0140-6736(21)01206-X 34509219

[B20] Cescon D. W. Bratman S. V. Chan S. M. Siu L. L. (2020). Circulating tumor DNA and liquid biopsy in oncology. Nat. Cancer 1 (3), 276–290. 10.1038/s43018-020-0043-5 35122035

[B21] Chang G. A. Tadepalli J. S. Shao Y. Zhang Y. Weiss S. Robinson E. (2016). Sensitivity of plasma BRAFmutant and NRASmutant cell-free DNA assays to detect metastatic melanoma in patients with low RECIST scores and non-RECIST disease progression. Mol. Oncol. 10 (1), 157–165. 10.1016/j.molonc.2015.09.005 26440707 PMC4695284

[B22] Chen G. Huang A. C. Zhang W. Zhang G. Wu M. Xu W. (2018). Exosomal PD-L1 contributes to immunosuppression and is associated with anti-PD-1 response. Nature 560 (7718), 382–386. 10.1038/s41586-018-0392-8 30089911 PMC6095740

[B23] Chen P.-S. Su J.-L. Hung M.-C. (2012). Dysregulation of MicroRNAs in cancer. J. Biomed. Sci. 19 (1), 90. 10.1186/1423-0127-19-90 23075324 PMC3482395

[B24] Chen Z. Li C. Zhou Y. Yao Y. Liu J. Wu M. (2020)2023). Liquid biopsies for cancer: from bench to clinic. MedComm. 4 (4), e329. 10.1002/mco2.329 PMC10363811 37492785

[B25] Cheng J. Nonaka T. Wong D. T. W. (2019). Salivary exosomes as nanocarriers for cancer biomarker delivery. Mater. (Basel) 12 (4), 654. 10.3390/ma12040654 PMC6416587 30795593

[B26] Cordonnier M. Nardin C. Chanteloup G. Derangere V. Algros M. P. Arnould L. (2020). Tracking the evolution of circulating exosomal-PD-L1 to monitor melanoma patients. J. Extracell. Vesicles 9 (1), 1710899. 10.1080/20013078.2019.1710899 32002173 PMC6968537

[B27] Diem S. Kasenda B. Martin-Liberal J. Lee A. Chauhan D. Gore M. (2015). Prognostic score for patients with advanced melanoma treated with ipilimumab. Eur. J. Cancer 51 (18), 2785–2791. 10.1016/j.ejca.2015.09.007 26597444

[B28] Elez E. Ros J. Fernandez J. Villacampa G. Moreno-Cardenas A. B. Arenillas C. (2022). RNF43 mutations predict response to anti-BRAF/EGFR combinatory therapies in BRAF(V600E) metastatic colorectal cancer. Nat. Med. 28 (10), 2162–2170. 10.1038/s41591-022-01976-z 36097219 PMC9556333

[B29] Esquela-Kerscher A. Slack F. J. (2006). Oncomirs - microRNAs with a role in cancer. Nat. Rev. Cancer 6 (4), 259–269. 10.1038/nrc1840 16557279

[B30] Felix J. Cassinat B. Porcher R. Schlageter M.-H. Maubec E. Pages C. (2016). Relevance of serum biomarkers associated with melanoma during follow-up of anti-CTLA-4 immunotherapy. Int. Immunopharmacol. 40, 466–473. 10.1016/j.intimp.2016.09.030 27728898

[B31] Fleming N. H. Zhong J. da Silva I. P. Vega-Saenz de Miera E. Brady B. Han S. W. (2015). Serum-based miRNAs in the prediction and detection of recurrence in melanoma patients. Cancer 121 (1), 51–59. 10.1002/cncr.28981 25155861 PMC4270907

[B32] Fogli S. Polini B. Carpi S. Pardini B. Naccarati A. Dubbini N. (2017). Identification of plasma microRNAs as new potential biomarkers with high diagnostic power in human cutaneous melanoma. Tumor Biol. 39 (5), 1010428317701646. 10.1177/1010428317701646 28466785

[B33] Foser S. Maiese K. Digumarthy S. R. Puig-Butille J. A. Rebhan C. (2024). Looking to the future of early detection in cancer: liquid biopsies, imaging, and artificial intelligence. Clin. Chem. 70 (1), 27–32. 10.1093/clinchem/hvad196 38175601

[B34] Freeman J. B. Gray E. S. Millward M. Pearce R. Ziman M. (2012). Evaluation of a multi-marker immunomagnetic enrichment assay for the quantification of circulating melanoma cells. J. Transl. Med. 10 (1), 192. 10.1186/1479-5876-10-192 22978632 PMC3480925

[B35] Gaiser M. R. von Bubnoff N. Gebhardt C. Utikal J. S. (2018). Liquid biopsy to monitor melanoma patients. JDDG J. der Deutschen Dermatologischen Gesellschaft. 16 (4), 405–414. 10.1111/ddg.13461 29512873

[B36] Garbe C. Hauschild A. Volkenandt M. Schadendorf D. Stolz W. Reinhold U. (2007). Evidence and interdisciplinary consense-based German guidelines: diagnosis and surveillance of melanoma. Melanoma Res. 17 (6), 393–399. 10.1097/CMR.0b013e3282f05039 17992123

[B37] Garbe C. Schadendorf D. Stolz W. Volkenandt M. Reinhold U. Kortmann R. D. (2008). Short German guidelines: malignant melanoma. J. Dtsch. Dermatol Ges. 6 (Suppl. 1), S9-S14–s14. 10.1111/j.1610-0387.2008.06711.x 18801142

[B38] Gershenwald J. E. Scolyer R. A. Hess K. R. Sondak V. K. Long G. V. Ross M. I. (2017). Melanoma staging: evidence-based changes in the American Joint Committee on Cancer eighth edition cancer staging manual. CA A Cancer J. Clin. 67 (6), 472–492. 10.3322/caac.21409 PMC5978683 29028110

[B39] Ginghina O. Hudita A. Zamfir M. Spanu A. Mardare M. Bondoc I. (2022). Liquid biopsy and artificial intelligence as tools to detect signatures of colorectal malignancies: a modern approach in patient's stratification. Front. Oncol. 12, 856575. 10.3389/fonc.2022.856575 35356214 PMC8959149

[B40] Gray E. S. Rizos H. Reid A. L. Boyd S. C. Pereira M. R. Lo J. (2015). Circulating tumor DNA to monitor treatment response and detect acquired resistance in patients with metastatic melanoma. Oncotarget 6 (39), 42008–42018. 10.18632/oncotarget.5788 26524482 PMC4747205

[B41] Greenberg E. Besser M. J. Ben-Ami E. Shapira-Frommer R. Itzhaki O. Zikich D. (2013). A comparative analysis of total serum miRNA profiles identifies novel signature that is highly indicative of metastatic melanoma: a pilot study. Biomarkers 18 (6), 502–508. 10.3109/1354750X.2013.816777 23902289

[B42] Guo S. Guo W. Li S. Dai W. Zhang N. Zhao T. (2016). Serum miR-16: a potential biomarker for predicting melanoma prognosis. J. Investigative Dermatology 136 (5), 985–993. 10.1016/j.jid.2015.12.041 26829037

[B43] Hamberg A. P. Korse C. M. Bonfrer J. M. de Gast G. C. (2003). Serum S100B is suitable for prediction and monitoring of response to chemoimmunotherapy in metastatic malignant melanoma. Melanoma Res. 13 (1), 45–49. 10.1097/00008390-200302000-00008 12569284

[B44] Hauschild A. Engel G. Brenner W. Gläser R. Mönig H. Henze E. (1999). S100B protein detection in serum is a significant prognostic factor in metastatic melanoma. Oncology 56 (4), 338–344. 10.1159/000011989 10343200

[B45] Heitzer E. Ulz P. Geigl J. B. (2015). Circulating tumor DNA as a liquid biopsy for cancer. Clin. Chem. 61 (1), 112–123. 10.1373/clinchem.2014.222679 25388429

[B46] Hong X. Sullivan R. J. Kalinich M. Kwan T. T. Giobbie-Hurder A. Pan S. (2018). Molecular signatures of circulating melanoma cells for monitoring early response to immune checkpoint therapy. Proc. Natl. Acad. Sci. 115 (10), 2467–2472. 10.1073/pnas.1719264115 29453278 PMC5877960

[B47] Hoon D. S. Bostick P. Kuo C. Okamoto T. Wang H. J. Elashoff R. (2000). Molecular markers in blood as surrogate prognostic indicators of melanoma recurrence. Cancer Res. 60 (8), 2253–2257. 10786692

[B48] Hoon D. S. Spugnardi M. Kuo C. Huang S. K. Morton D. L. Taback B. (2004). Profiling epigenetic inactivation of tumor suppressor genes in tumors and plasma from cutaneous melanoma patients. Oncogene 23 (22), 4014–4022. 10.1038/sj.onc.1207505 15064737 PMC2856469

[B49] Hu C. Jiang W. Lv M. Fan S. Lu Y. Wu Q. (2022). Potentiality of exosomal proteins as novel cancer biomarkers for liquid biopsy. Front. Immunol. 13, 792046. 10.3389/fimmu.2022.792046 35757760 PMC9218252

[B50] Huang S. K. Hoon D. S. B. (2016). Liquid biopsy utility for the surveillance of cutaneous malignant melanoma patients. Mol. Oncol. 10 (3), 450–463. 10.1016/j.molonc.2015.12.008 26778792 PMC5307330

[B51] Ignatiadis M. Sledge G. W. Jeffrey S. S. (2021). Liquid biopsy enters the clinic - implementation issues and future challenges. Nat. Rev. Clin. Oncol. 18 (5), 297–312. 10.1038/s41571-020-00457-x 33473219

[B52] Ilie M. Benzaquen J. Hofman V. Lassalle S. Yazbeck N. Leroy S. (2017). Immunotherapy in non-small cell lung cancer: biological principles and future opportunities. Curr. Mol. Med. 17 (8), 527–540. 10.2174/1566524018666180222114038 29473504

[B53] Jahr S. Hentze H. Englisch S. Hardt D. Fackelmayer F. O. Hesch R.-D. (2001). DNA fragments in the blood plasma of cancer patients: quantitations and evidence for their origin from apoptotic and necrotic cells. Cancer Res. 61 (4), 1659–1665. 11245480

[B54] Ji Z. Njauw C. N. Guhan S. Kumar R. Reddy B. Rajadurai A. (2021). Loss of ACK1 upregulates EGFR and mediates resistance to BRAF inhibition. J. Investig. Dermatol. 141 (5), 1317–1324.e1. 10.1016/j.jid.2020.06.041 33159968

[B55] Jones N. Nonaka T. (2024). Circulating miRNAs as biomarkers for the diagnosis in patients with melanoma: systematic review and meta-analysis. Front. Genet. 15, 1339357. 10.3389/fgene.2024.1339357 38419786 PMC10899317

[B56] Kamińska P. Buszka K. Zabel M. Nowicki M. Alix-Panabières C. Budna-Tukan J. (2021). Liquid biopsy in melanoma: significance in diagnostics, prediction and treatment monitoring. Int. J. Mol. Sci. 22 (18), 9714. 10.3390/ijms22189714 34575876 PMC8468624

[B57] Kanemaru H. Fukushima S. Yamashita J. Honda N. Oyama R. Kakimoto A. (2011). The circulating microRNA-221 level in patients with malignant melanoma as a new tumor marker. J. Dermatological Sci. 61 (3), 187–193. 10.1016/j.jdermsci.2010.12.010 21273047

[B58] Karnell R. Schoultz E. V. Hansson L.-O. Nilsson B. Arstrand K. Kagedal B. (1997). S100B protein, 5-S-cysteinyldopa and 6-hydroxy-5- methoxyindole-2-carboxylic acid as biochemical markers for survival prognosis in patients with malignant melanoma. Melanoma Res. 7 (5), 393–399. 10.1097/00008390-199710000-00005 9429222

[B59] Kasi P. M. Chakrabarti S. Sawyer S. Krainock M. Poklepovic A. Ansstas G. (2022). BESPOKE IO protocol: a multicentre, prospective observational study evaluating the utility of ctDNA in guiding immunotherapy in patients with advanced solid tumours. BMJ Open 12 (5), e060342. 10.1136/bmjopen-2021-060342 PMC9152946 35636789

[B60] Khattak M. A. Reid A. Freeman J. Pereira M. McEvoy A. Lo J. (2020). PD-L1 expression on circulating tumor cells may Be predictive of response to pembrolizumab in advanced melanoma: results from a pilot study. Oncologist 25 (3), e520–e527. 10.1634/theoncologist.2019-0557 32162809 PMC7066715

[B61] Klinac D. Gray E. S. Freeman J. B. Reid A. Bowyer S. Millward M. (2014). Monitoring changes in circulating tumour cells as a prognostic indicator of overall survival and treatment response in patients with metastatic melanoma. BMC Cancer 14 (1), 423. 10.1186/1471-2407-14-423 24915896 PMC4060872

[B62] Kong D. Zhang W. Yang Z. Li G. Cheng S. Zhang K. (2021). Correlation between PD-L1 expression ON CTCs and prognosis of patients with cancer: a systematic review and meta-analysis. Oncoimmunology 10 (1), 1938476. 10.1080/2162402X.2021.1938476 34211802 PMC8218685

[B63] Lee J. H. Long G. V. Boyd S. Lo S. Menzies A. M. Tembe V. (2017). Circulating tumour DNA predicts response to anti-PD1 antibodies in metastatic melanoma. Ann. Oncol. 28 (5), 1130–1136. 10.1093/annonc/mdx026 28327969

[B64] Li P. He Q. Y. Luo C. Q. Qian L. Y. (2014). Circulating miR-221 expression level and prognosis of cutaneous malignant melanoma. Med. Sci. Monit. 20, 2472–2477. 10.12659/MSM.891327 25430553 PMC4259523

[B65] Li P. Liu S. Du L. Mohseni G. Zhang Y. Wang C. (2022). Liquid biopsies based on DNA methylation as biomarkers for the detection and prognosis of lung cancer. Clin. Epigenetics 14 (1), 118. 10.1186/s13148-022-01337-0 36153611 PMC9509651

[B66] Lin J. Yang Q. Wilder P. T. Carrier F. Weber D. J. (2010). The calcium-binding protein S100B down-regulates p53 and apoptosis in malignant melanoma. J. Biol. Chem. 285 (35), 27487–27498. 10.1074/jbc.M110.155382 20587415 PMC2930747

[B67] Lin S. Y. Orozco J. I. J. Hoon D. S. B. (2018). Detection of minimal residual disease and its clinical applications in melanoma and breast cancer patients. Adv. Exp. Med. Biol. 1100, 83–95. 10.1007/978-3-319-97746-1_5 30411261

[B68] Long G. V. Grob J.-J. Nathan P. Ribas A. Robert C. Schadendorf D. (2016). Factors predictive of response, disease progression, and overall survival after dabrafenib and trametinib combination treatment: a pooled analysis of individual patient data from randomised trials. Lancet Oncol. 17 (12), 1743–1754. 10.1016/S1470-2045(16)30578-2 27864013

[B69] Lucci A. Hall C. S. Patel S. P. Narendran B. Bauldry J. B. Royal R. E. (2020). Circulating tumor cells and early relapse in node-positive melanoma. Clin. Cancer Res. 26 (8), 1886–1895. 10.1158/1078-0432.CCR-19-2670 32015020

[B70] Marczynski G. T. Laus A. C. dos Reis M. B. Reis R. M. Vazquez V. L. (2020). Circulating tumor DNA (ctDNA) detection is associated with shorter progression-free survival in advanced melanoma patients. Sci. Rep. 10 (1), 18682. 10.1038/s41598-020-75792-1 33122747 PMC7596487

[B71] Margue C. Reinsbach S. Philippidou D. Beaume N. Walters C. Schneider J. G. (2015). Comparison of a healthy miRNome with melanoma patient miRNomes: are microRNAs suitable serum biomarkers for cancer? Oncotarget 6 (14), 12110–12127. 10.18632/oncotarget.3661 25883223 PMC4494926

[B72] Marsavela G. Aya-Bonilla C. A. Warkiani M. E. Gray E. S. Ziman M. (2018). Melanoma circulating tumor cells: benefits and challenges required for clinical application. Cancer Lett. 424, 1–8. 10.1016/j.canlet.2018.03.013 29548820

[B73] Marzese D. M. Hirose H. Hoon D. S. B. (2013). Diagnostic and prognostic value of circulating tumor-related DNA in cancer patients. Expert Rev. Mol. Diagnostics 13 (8), 827–844. 10.1586/14737159.2013.845088 24127721

[B74] Matthews N. H. Li W.-Q. Qureshi A. A. Weinstock M. A. Cho E. Codon publications; 2017:3–22. 29461782

[B75] Mattox A. K. Bettegowda C. Zhou S. Papadopoulos N. Kinzler K. W. Vogelstein B. (2019). Applications of liquid biopsies for cancer. Sci. Transl. Med. 11 (507), eaay1984. 10.1126/scitranslmed.aay1984 31462507

[B76] Millet A. Martin A. R. Ronco C. Rocchi S. Benhida R. (2017). Metastatic melanoma: insights into the evolution of the treatments and future challenges. Med. Res. Rev. 37 (1), 98–148. 10.1002/med.21404 27569556

[B77] Mitchell P. S. Parkin R. K. Kroh E. M. Fritz B. R. Wyman S. K. Pogosova-Agadjanyan E. L. (2008). Circulating microRNAs as stable blood-based markers for cancer detection. Proc. Natl. Acad. Sci. 105 (30), 10513–10518. 10.1073/pnas.0804549105 18663219 PMC2492472

[B78] Mohammadpour A. Derakhshan M. Darabi H. Hedayat P. Momeni M. (2019). Melanoma: where we are and where we go. J. Cell. Physiol. 234 (4), 3307–3320. 10.1002/jcp.27286 30362507

[B79] Mori T. Martinez S. R. O'Day S. J. Morton D. L. Umetani N. Kitago M. (2006). Estrogen receptor-alpha methylation predicts melanoma progression. Cancer Res. 66 (13), 6692–6698. 10.1158/0008-5472.CAN-06-0801 16818643 PMC2856460

[B80] Morrissey S. M. Zhang F. Ding C. Montoya-Durango D. E. Hu X. Yang C. (2021). Tumor-derived exosomes drive immunosuppressive macrophages in a pre-metastatic niche through glycolytic dominant metabolic reprogramming. Cell. Metab. 33 (10), 2040–2058.e10. 10.1016/j.cmet.2021.09.002 34559989 PMC8506837

[B81] Mustachio L. M. Chelariu-Raicu A. Szekvolgyi L. Roszik J. (2021). Targeting KRAS in cancer: promising therapeutic strategies. Cancers (Basel) 13 (6), 1204. 10.3390/cancers13061204 33801965 PMC7999304

[B82] Nakamura T. Sueoka-Aragane N. Iwanaga K. Sato A. Komiya K. Abe T. (2011). A noninvasive system for monitoring resistance to epidermal growth factor receptor tyrosine kinase inhibitors with plasma DNA. J. Thorac. Oncol. 6 (10), 1639–1648. 10.1097/JTO.0b013e31822956e8 21921847

[B83] Nikolin B. Djan I. Trifunovic J. Dugandzija T. Novkovic D. Djan V. (2016). MIA, S100 and LDH as important predictors of overall survival of patients with stage IIb and IIc melanoma. J. buon 21 (3), 691–697. 27569092

[B84] Nonaka T. Wong D. T. W. (2017). Saliva-Exosomics in cancer: molecular characterization of cancer-derived exosomes in saliva. Enzymes 42, 125–151. 10.1016/bs.enz.2017.08.002 29054268 PMC6167313

[B85] Nonaka T. Wong D. T. W. (2018). Liquid biopsy in head and neck cancer: promises and challenges. J. Dent. Res. 97 (6), 701–708. 10.1177/0022034518762071 29513618 PMC5960882

[B86] Nonaka T. Wong D. T. W. (2022). Saliva diagnostics. Annu. Rev. Anal. Chem. Palo Alto Calif. 15 (1), 107–121. 10.1146/annurev-anchem-061020-123959 35696523 PMC9348814

[B87] Nonaka T. Wong D. T. W. (2023). Saliva diagnostics: salivaomics, saliva exosomics, and saliva liquid biopsy. J. Am. Dent. Assoc. 154 (8), 696–704. 10.1016/j.adaj.2023.05.006 37500232 PMC11390004

[B88] Palmer S. R. Erickson L. A. Ichetovkin I. Knauer D. J. Markovic S. N. (2011). Circulating serologic and molecular biomarkers in malignant melanoma. Mayo Clin. Proc. 86 (10), 981–990. 10.4065/mcp.2011.0287 21964175 PMC3184027

[B89] Pantel K. Alix-Panabieres C. (2019). Liquid biopsy and minimal residual disease - latest advances and implications for cure. Nat. Rev. Clin. Oncol. 16 (7), 409–424. 10.1038/s41571-019-0187-3 30796368

[B90] Pastwinska J. Karas K. Karwaciak I. Ratajewski M. (2022). Targeting EGFR in melanoma - the sea of possibilities to overcome drug resistance. Biochim. Biophys. Acta Rev. Cancer 1877 (4), 188754. 10.1016/j.bbcan.2022.188754 35772580

[B91] Patel R. P. Somasundram P. M. Smith L. K. Sheppard K. E. McArthur G. A. (2023). The therapeutic potential of targeting minimal residual disease in melanoma. Clin. Transl. Med. 13 (3), e1197. 10.1002/ctm2.1197 36967556 PMC10040726

[B92] Peinado H. Alečković M. Lavotshkin S. Matei I. Costa-Silva B. Moreno-Bueno G. (2012). Melanoma exosomes educate bone marrow progenitor cells toward a pro-metastatic phenotype through MET. Nat. Med. 18 (6), 883–891. 10.1038/nm.2753 22635005 PMC3645291

[B93] Raimondi L. De Luca A. Costa V. Amodio N. Carina V. Bellavia D. (2017). Circulating biomarkers in osteosarcoma: new translational tools for diagnosis and treatment. Oncotarget 8 (59), 100831–100851. 10.18632/oncotarget.19852 29246026 PMC5725068

[B94] Reynolds S. R. Albrecht J. Shapiro R. L. Roses D. F. Harris M. N. Conrad A. (2003). Changes in the presence of multiple markers of circulating melanoma cells correlate with clinical outcome in patients with melanoma. Clin. Cancer Res. 9 (4), 1497–1502. 12684425

[B95] Sacco A. Forgione L. Carotenuto M. De Luca A. Ascierto P. A. Botti G. (2020). Circulating tumor DNA testing opens new perspectives in melanoma management. Cancers 12 (10), 2914. 10.3390/cancers12102914 33050536 PMC7601606

[B96] Sahranavardfard P. Firouzi J. Azimi M. Khosravani P. Heydari R. Emami Razavi A. (2019). MicroRNA-203 reinforces stemness properties in melanoma and augments tumorigenesis *in vivo* . J. Cell. Physiology 234 (11), 20193–20205. 10.1002/jcp.28619 31016725

[B97] Salama I. Malone P. S. Mihaimeed F. Jones J. L. (2008). A review of the S100 proteins in cancer. Eur. J. Surg. Oncol. 34 (4), 357–364. 10.1016/j.ejso.2007.04.009 17566693

[B98] Samija I. Lukac J. Marić-Brozić J. Buljan M. Alajbeg I. Kovacević D. (2010). Prognostic value of microphthalmia-associated transcription factor and tyrosinase as markers for circulating tumor cells detection in patients with melanoma. Melanoma Res. 20 (4), 293–302. 10.1097/CMR.0b013e32833906b6 20357686

[B99] Sanmamed M. F. Fernandez-Landazuri S. Rodriguez C. Lozano M. D. Echeveste J. I. Perez G. J. L. (2014). Relevance of MIA and S100 serum tumor markers to monitor BRAF inhibitor therapy in metastatic melanoma patients. Clin. Chim. Acta 429, 168–174. 10.1016/j.cca.2013.11.034 24333389

[B100] Sanmamed M. F. Fernández-Landázuri S. Rodríguez C. Zárate R. Lozano M. D. Zubiri L. (2015). Quantitative cell-free circulating BRAFV600E mutation analysis by use of droplet digital PCR in the follow-up of patients with melanoma being treated with BRAF inhibitors. Clin. Chem. 61 (1), 297–304. 10.1373/clinchem.2014.230235 25411185

[B101] Santiago-Walker A. Gagnon R. Mazumdar J. Casey M. Long G. V. Schadendorf D. (2016). Correlation of BRAF mutation status in circulating-free DNA and tumor and association with clinical outcome across four BRAFi and MEKi clinical trials. Clin. Cancer Res. 22 (3), 567–574. 10.1158/1078-0432.CCR-15-0321 26446943

[B102] Scholer L. V. Reinert T. Orntoft M. W. Kassentoft C. G. Arnadottir S. S. Vang S. (2017). Clinical implications of monitoring circulating tumor DNA in patients with colorectal cancer. Clin. Cancer Res. 23 (18), 5437–5445. 10.1158/1078-0432.CCR-17-0510 28600478

[B103] Schultz E. S. Diepgen T. L. Von Den Driesch P. (1998). Clinical and prognostic relevance of serum S-100 beta protein in malignant melanoma. Br. J. Dermatol 138 (3), 426–430. 10.1046/j.1365-2133.1998.02119.x 9580794

[B104] Simiczyjew A. Pietraszek-Gremplewicz K. Dratkiewicz E. Podgorska M. Matkowski R. Zietek M. (2019). Combination of selected MET and EGFR inhibitors decreases melanoma cells' invasive abilities. Front. Pharmacol. 10, 1116. 10.3389/fphar.2019.01116 31649529 PMC6792435

[B105] Siravegna G. Marsoni S. Siena S. Bardelli A. (2017). Integrating liquid biopsies into the management of cancer. Nat. Rev. Clin. Oncol. 14 (9), 531–548. 10.1038/nrclinonc.2017.14 28252003

[B106] Siravegna G. Mussolin B. Buscarino M. Corti G. Cassingena A. Crisafulli G. (2015). Clonal evolution and resistance to EGFR blockade in the blood of colorectal cancer patients. Nat. Med. 21 (7), 795–801. 10.1038/nm.3870 26030179 PMC4868598

[B107] Sparano J. O'Neill A. Alpaugh K. Wolff A. C. Northfelt D. W. Dang C. T. (2018). Association of circulating tumor cells with late recurrence of estrogen receptor-positive breast cancer: a secondary analysis of a randomized clinical trial. JAMA Oncol. 4 (12), 1700–1706. 10.1001/jamaoncol.2018.2574 30054636 PMC6385891

[B108] Stark M. S. Klein K. Weide B. Haydu L. E. Pflugfelder A. Tang Y. H. (2015). The prognostic and predictive value of melanoma-related MicroRNAs using tissue and serum: a MicroRNA expression analysis. eBioMedicine 2 (7), 671–680. 10.1016/j.ebiom.2015.05.011 26288839 PMC4534690

[B109] Strazzulla L. C. Li X. Zhu K. Okhovat J.-P. Lee S. J. Kim C. C. (2019). Clinicopathologic, misdiagnosis, and survival differences between clinically amelanotic melanomas and pigmented melanomas. J. Am. Acad. Dermatology 80 (5), 1292–1298. 10.1016/j.jaad.2019.01.012 30654075

[B110] Sun Y. Hou Z. Luo B. Li C. Liu J. Liu J. (2021). Circular RNA circRNA_0082835 promotes progression and lymphatic metastasis of primary melanoma by sponging microRNA miRNA-429. Bioengineered 12 (1), 4159–4173. 10.1080/21655979.2021.1953822 34288815 PMC8806410

[B111] Tarhini A. A. Stuckert J. Lee S. Sander C. Kirkwood J. M. (2009). Prognostic significance of serum S100B protein in high-risk surgically resected melanoma patients participating in Intergroup Trial ECOG 1694. J. Clin. Oncol. 27 (1), 38–44. 10.1200/JCO.2008.17.1777 19047287 PMC3426933

[B112] Thierry A. R. El Messaoudi S. Gahan P. B. Anker P. Stroun M. (2016). Origins, structures, and functions of circulating DNA in oncology. Cancer Metastasis Rev. 35 (3), 347–376. 10.1007/s10555-016-9629-x 27392603 PMC5035665

[B113] Tian R. Liu T. Qiao L. Gao M. Li J. (2015). Decreased serum microRNA-206 level predicts unfavorable prognosis in patients with melanoma. Int. J. Clin. Exp. Pathol. 8 (3), 3097–3103. 26045823 PMC4440132

[B114] Tie J. Wang Y. Tomasetti C. Li L. Springer S. Kinde I. (2016). Circulating tumor DNA analysis detects minimal residual disease and predicts recurrence in patients with stage II colon cancer. Sci. Transl. Med. 8 (346), 346ra92. 10.1126/scitranslmed.aaf6219 PMC5346159 27384348

[B115] Trapp E. Janni W. Schindlbeck C. Juckstock J. Andergassen U. de Gregorio A. (2019). Presence of circulating tumor cells in high-risk early breast cancer during follow-up and prognosis. J. Natl. Cancer Inst. 111 (4), 380–387. 10.1093/jnci/djy152 30312434

[B116] Ulmer A. Schmidt-Kittler O. Fischer J. Ellwanger U. Rassner G. Riethmüller G. (2004). Immunomagnetic enrichment, genomic characterization, and prognostic impact of circulating melanoma cells. Clin. Cancer Res. 10 (2), 531–537. 10.1158/1078-0432.ccr-0424-03 14760074

[B117] Van Laar R. Latif B. King S. Love C. Taubenheim N. Kalansooriya E. (2023). Validation of a microRNA liquid biopsy assay for diagnosis and risk stratification of invasive cutaneous melanoma. Br. J. Dermatol 189 (3), 292–301. 10.1093/bjd/ljad137 37144735

[B118] Van Laar R. Lincoln M. Van Laar B. (2018). Development and validation of a plasma-based melanoma biomarker suitable for clinical use. Br. J. Cancer 118 (6), 857–866. 10.1038/bjc.2017.477 29360813 PMC5886119

[B119] von Schoultz E. Hansson L. O. Djureen E. Hansson J. Kärnell R. Nilsson B. (1996). Prognostic value of serum analyses of S-100β protein in malignant melanoma. Melanoma Res. 6 (2), 133–138. 8791271 10.1097/00008390-199604000-00008

[B120] Wagner N. B. Forschner A. Leiter U. Garbe C. Eigentler T. K. (2018). S100B and LDH as early prognostic markers for response and overall survival in melanoma patients treated with anti-PD-1 or combined anti-PD-1 plus anti-CTLA-4 antibodies. Br. J. Cancer 119 (3), 339–346. 10.1038/s41416-018-0167-x 29950611 PMC6070917

[B121] Wang X. Xia J. Yang L. Dai J. He L. (2023). Recent progress in exosome research: isolation, characterization and clinical applications. Cancer Gene Ther. 30 (8), 1051–1065. 10.1038/s41417-023-00617-y 37106070

[B122] Wang Z. Chen J.-Q. Liu J.-l. Tian L. (2016). Exosomes in tumor microenvironment: novel transporters and biomarkers. J. Transl. Med. 14 (1), 297. 10.1186/s12967-016-1056-9 27756426 PMC5070309

[B123] Webber J. Steadman R. Mason M. D. Tabi Z. Clayton A. (2010). Cancer exosomes trigger fibroblast to myofibroblast differentiation. Cancer Res. 70 (23), 9621–9630. 10.1158/0008-5472.CAN-10-1722 21098712

[B124] Welsh S. J. Rizos H. Scolyer R. A. Long G. V. (2016). Resistance to combination BRAF and MEK inhibition in metastatic melanoma: where to next? Eur. J. Cancer 62, 76–85. 10.1016/j.ejca.2016.04.005 27232329

[B125] Wong S. Q. Raleigh J. M. Callahan J. Vergara I. A. Ftouni S. Hatzimihalis A. (2017). Circulating tumor DNA analysis and functional imaging provide complementary approaches for comprehensive disease monitoring in metastatic melanoma. JCO Precis. Oncol. 1 (1), 1–14. 10.1200/PO.16.00009 35172485

[B126] Xi L. Nicastri D. G. El-Hefnawy T. Hughes S. J. Luketich J. D. Godfrey T. E. (2007). Optimal markers for real-time quantitative reverse transcription PCR detection of circulating tumor cells from melanoma, breast, colon, esophageal, head and neck, and lung cancers. Clin. Chem. 53 (7), 1206–1215. 10.1373/clinchem.2006.081828 17525108

[B127] Xiao D. Ohlendorf J. Chen Y. Taylor D. D. Rai S. N. Waigel S. (2012). Identifying mRNA, MicroRNA and protein profiles of melanoma exosomes. PLOS ONE 7 (10), e46874. 10.1371/journal.pone.0046874 23056502 PMC3467276

[B128] Yancovitz M. Litterman A. Yoon J. Ng E. Shapiro R. L. Berman R. S. (2012). Intra- and inter-tumor heterogeneity of BRAFV600EMutations in primary and metastatic melanoma. PLOS ONE 7 (1), e29336. 10.1371/journal.pone.0029336 22235286 PMC3250426

[B129] Yáñez-Mó M. Siljander P. R. M. Andreu Z. Bedina Zavec A. Borràs F. E. Buzas E. I. (2015). Biological properties of extracellular vesicles and their physiological functions. J. Extracell. Vesicles 4 (1), 27066. 10.3402/jev.v4.27066 25979354 PMC4433489

[B130] Yang C. Xia B. R. Jin W. L. Lou G. (2019). Circulating tumor cells in precision oncology: clinical applications in liquid biopsy and 3D organoid model. Cancer Cell. Int. 19, 341. 10.1186/s12935-019-1067-8 31866766 PMC6918690

[B131] Ye J. Xu M. Tian X. Cai S. Zeng S. (2019). Research advances in the detection of miRNA. J. Pharm. Analysis 9 (4), 217–226. 10.1016/j.jpha.2019.05.004 PMC6702429 31452959

[B132] Ye L. F. Huang Z. Y. Chen X. X. Chen Z. G. Wu S. X. Ren C. (2022). Monitoring tumour resistance to the BRAF inhibitor combination regimen in colorectal cancer patients via circulating tumour DNA. Drug Resist Updat 65, 100883. 10.1016/j.drup.2022.100883 36202008

[B133] Zhang C. Fan Y. Che X. Zhang M. Li Z. Li C. (2020). Anti-PD-1 therapy response predicted by the combination of exosomal PD-L1 and CD28. Front. Oncol. 10, 760. 10.3389/fonc.2020.00760 32528882 PMC7266952

[B134] Zhou Y. Kuang Y. Wang C. Yu Y. Pan L. Hu X. (2024). Impact of KRAS mutation on the tumor microenvironment in colorectal cancer. Int. J. Biol. Sci. 20 (5), 1947–1964. 10.7150/ijbs.88779 38481800 PMC10929194

